# Enpp1 ameliorates MAFLD by regulating hepatocyte lipid metabolism through the AMPK/PPARα signaling pathway

**DOI:** 10.1186/s13578-025-01364-3

**Published:** 2025-02-19

**Authors:** Xiaohui Liu, Shuai Chen, Xing Liu, Xianxian Wu, Xiaoliang Jiang, Yuhan Li, Zhiwei Yang

**Affiliations:** 1 Institute of Laboratory Animal Science, Chinese Academy of Medical Sciences (CAMS) & Comparative Medicine Centre, Peking Union Medical College (PUMC), Beijing, 100021 China; 2 Fuyang People’s Hospital affiliated to Anhui Medical University, Fuyang, China; 3 Department of Clinical Laboratory, Beijing Friendship Hospital, Capital Medical University, Beijing, 100050 China

**Keywords:** Metabolic dysfunction-associated fatty liver disease, Enpp1, Lipid metabolism, AMPK, PPARα

## Abstract

**Background:**

Metabolic dysfunction-associated fatty liver disease (MAFLD) has become the leading chronic liver disease globally, and there are no approved pharmacotherapies to treat this disease. Ectonucleotide pyrophosphatase/phosphodiesterase 1 (Enpp1) has been found to be related to insulin resistance and lipid accumulation. However, the role and mechanism of Enpp1 in the development of MAFLD remain unknown.

**Results:**

Here we discovered that Enpp1 is lowly expressed in the liver of MAFLD patients by clinical investigation. Knocking out Enpp1 in the liver of mice aggravated obesity, insulin resistance and hepatic steatosis, and these effects were reversed by liver-specific Enpp1 overexpression. Through transcriptomic data mining and experimental validation, we demonstrated that Enpp1 deficiency inhibited the expression of AMPK (energy receptor) and PPARα (nuclear transcription factor for lipid metabolism), thereby promoting the transcription of lipid synthesis factors and mediating the progression of MAFLD. Mechanistically, Enpp1 enhanced the activity of AMPK by increasing the AMP-to-ATP ratio, which in turn raised PPARα levels and promoted the transcription of its downstream lipid metabolism factors. Pharmacological inhibition of AMPK activity abolished the promoting effect of Enpp1 on PPARα protein expression.

**Conclusions:**

This study indicate that Enpp1 can effectively ameliorate MAFLD through effects on AMPK/PPARα signaling pathway-mediated lipid metabolism, revealing the significance of Enpp1 as a promising therapeutic target against MAFLD.

**Supplementary Information:**

The online version contains supplementary material available at 10.1186/s13578-025-01364-3.

## Introduction

Metabolic dysfunction-associated fatty liver disease (MAFLD) is a disease that presents with significant accumulation of fats in the liver without history excessive alcohol intake and other risk factors of liver fat accumulation. It can progress to metabolic dysfunction-associated steatohepatitis (MASH), which contributes to the occurrence of liver fibrosis, cirrhosis and hepatocellular carcinoma [1, 2]. MAFLD has become the most prevalent chronic liver disease globally, with a prevalence of 25% of the global population, and a notable increase in prevalence among adolescents and children [3]. However, there is still no effective treatment for this chronic liver disease, highlighting the urgent need explore new therapeutic targets. Insulin resistance, an early indicator of various metabolic disorders, has been identified as a key factor associated with the accmulation of liver fat [4]. This is largely attributed to the reduced ability of insulin to suppress the breakdown of fat in adipose tissue, resulting in an increased flow of free fatty acids to the liver and the synthesis of triglycerides, potentially exacerbating hepatic steatosis [5, 6]. This suggest that novel therapeutic targets that may elevate insulin sensitivity and modulate lipid metabolism need to be explored for effective MAFLD management.

Ectonucleotide pyrophosphatase/phosphodiesterase 1 (Enpp1), a type II transmembrane metalloenzyme, hydrolyzes extracellular ATP and GTP to form AMP and GMP, making a crucial modulator of mineralization in skeletal and soft tissues [7]. Previous studies on solid tumors including glioblastoma, ovarian, and breast have demonstrated overexpression of Enpp1 in these cancers [8–10]. It has been reported that Enpp1 promotes the chemotactic infiltration of polymorphonuclear marrow-derived inhibitory cells and suppress the tumor-infiltrating cytotoxic T cells preventing anti-tumor immune attack on breast cancer cells [10]. Evidence for its role in multiple metabolic diseases including diabetes, cardiovascular disease and MAFLD has been documented [7, 11, 12]. Some Enpp1 121Gln polymorphisms are associated with higher risk of hyperlipidemia and liver injury in patients with MAFLD with other scholars showing that Enpp1 aggravates insulin resistance [13]. Researchers have demonstrated that Enpp1 can participate in the regulation of lipid metabolism via inhibiting adipocyte maturation by down-regulating the expression of adipogenic genes [14]. Based on above findings, we speculate the Enpp1 can protect against MAFLD via modulating metabolic disorders.

In this study, a comprehensive analysis of the Enpp1 expression in MAFLD was performed. In addition, the effects of Enpp1 on the development of obesity and insulin resistance in MAFLD mice were explored, focusing on mechanisms associated with liver lipid accumulation. It was observed that the liver Enpp1 expression were lower in MAFLD patients and MAFLD mouse models, whereas the Enpp1 deficient MAFLD mice exhibited enhanced obesity, insulin resistance and hepatic steatosis. To elucidate the molecular mechanisms by which Enpp1 regulates lipid metabolism in MALFD liver, transcriptomic data of MAFLD mouse liver were screened which led to the identification of the AMPK/PPARα as a target of Enpp1. Considering that the AMPK/PPARα axis regulates liver lipid metabolism, we performed in vivo and *vitro* experiments to confirm that Enpp1 promotes the AMPK phosphorylation and promotes PPARα expression, inhibit the transcription of liver lipid synthesis genes and improve lipid accumulation in MAFLD liver. Additionally, our results indicated that the treatment of compound C, a small-molecule inhibitor of AMPK enzymatic activity, abolished the positive effects of Enpp1 on PPARα protein expression. These findings emphasise the role of Enpp1 in alleviating MAFLD progression by up-regulating AMPK/PPARα expression. Therefore, we elucidated a novel mechanism by which Enpp1 regulates liver lipid metabolism in the liver, providing a new therapeutic strategy for MAFLD.

## Methods

### Human liver samples

Human liver biopsy specimens were obtained from 11 patients diagnosed with gallstones at the Ningxia Medical University Second Affiliated Hospital (Yinchuan, China). And then they were divided into an MAFLD group (*n* = 5) and an MAFLD control group (Non-MALFD, *n* = 6) based on ultrasound and histopathological analysis of the liver biopsy samples. This study was approved by the Institutional Ethics Committee of Ningxia Medical University and Ningxia Medical University Second Affiliated Hospital (approval number: 2019 − 228). All research was conducted in accordance with both the Declarations of Helsinki and Istanbul. Written consent was provided by all subjects. The steatosis indices of these liver specimens were accessed using haematoxylin and eosin (H&E) and Oil Red O staining.

### Animal experiments

All institutional and national guidelines for the care and use of laboratory animals were followed. All the animal experiments were approved by the Animal Care and Use Committee of the Institute of Experimental Animals, Chinese Academy of Medical Sciences and Beijing Union Medical College (approval number: yzw22002). All experiments were conducted using male mice. Liver-specific Enpp1 knockout (*Enpp1*^*fl/fl*^*-Alb-Cre*, CKO) mice were developed by Beijing Viewsolid Biotech, by crossing a male *Alb-Cre*^*+/−*^ mouse to a female *Enpp1*^*fl/fl*^ mouse. The db/db mice (genetic model of MAFLD) were also purchased from Beijing Viewsolid Biotech. CKO mice and controls were fed with a normal chow diet (NCD) or a high-fat diet (HFD: 60% calories from fat, 0.28% from cholesterol, and 20% calories from carbohydrate; Research Diets catalog MD12033, Medi-science, China) for 16 weeks.

For the overexpression of hepatic Enpp1, C57BL/6J male mice (8 weeks of age) were maintained on an HFD for 8 weeks and then injected with an Enpp1 liver-specific overexpression adenovirus (AAV8·Enpp1, 2 × 10^12^ genome copies) purchased from Vigene Bioscience. The mice were maintained on an HFD diet for another 8 weeks. Mice were then fasted for 6 h and anesthetized, after which serum, liver tissue, and adipose tissue from the epididymis were collected.

### Serum enzyme and lipid assays

All groups of mice were fasted for 6 h and then anesthetised for enucleation to collect serum samples. A biochemical analyser ((Mindray, China) was used to measure the serum levels of the liver enzymes alanine transaminase (ALT) and aspartate transaminase (AST), and the serum lipid contents of the triglyceride (TG), total cholesterol (T-CHO), low-density lipoprotein cholesterol (LDL-C) according to the manufacturer’s instructions. In liver tissues and cells, the TG content was measured using a triglyceride assay kit (A110-1-11, Nanjing Jiancheng) and the liver total cholesterol (T-CHO) was measured by a total cholesterol assay kit (A111-1-11, Nanjing Jiancheng). The results were normalised to the protein concentration.

### Intravenous glucose tolerance test (IGTT) and insulin tolerance test (ITT)

Glucose tolerance tests and insulin tolerance tests were conducted using intraperitoneal injections of glucose (2 mg/g) or insulin (0.75 IU/10 g), respectively, in mice that had been fasted for 6 h. Blood glucose levels were determined through tail bleeding (Sanocare Stable Non-adjustable Blood Glucose Test Strips, China) at predetermined intervals (0, 30, 60, 90, and 120 min). The area under the curve (AUC) was calculated using summation of trapezoids.

### Western blotting

Cells and liver tissues were lysed with RIPA lysis buffer containing a protease inhibitor and a phosphatase inhibitor. Subsequently, the protein lysis solution was centrifuged and the protein concentration was measured in the resulting supernatant. The protein samples were separated via 10% sodium dodecyl sulphate-polyacrylamide gel electrophoresis and transferred onto nitrocellulose membranes. The following antibodies were used in this study including anti-Enpp1 (1:2000, #2061S, Cell Signaling Technology), anti-phospho-AMPK (Thr172) (1:2000, #50081S, Cell Signaling Technology), anti-AMPK (1:2000, #2532S, Cell Signaling Technology), anti-PPARα (1:2000, #15540-1-AP, Proteintech), HRP-conjugated anti-β-actin antibodies (1:5000, #KC-5A08, KANGCHEN) and HRP-conjugated anti-rabbit IgG antibodies (1:10000, #b31402, Thermo Fisher Scientific).

### Quantitative polymerase chain reaction (qPCR)

Total RNA was extracted from liver tissue using a Total RNA Isolation Kit (#RC112-01, Vazyme) and was subsequently reverse-transcribed into cDNA via a reverse transcription reagent kit. qPCR was performed using a SYBR Green PCR Master Mix (#Q311-02, Vazyme) according to the manufacturer’s protocol. Relative changes in mRNA expression were calculated using the 2^−ΔΔCt^ method. The sequences of primers used are listed in Table S1.

### Oil red O staining

Fresh liver tissues were submerged in a 30% sucrose solution at 4 °C for dehydration. Subsequently, the tissues were excised into 8 μm thick frozen section, and Oil red O staining was performed using an Oil red O stain kit (#G1261, Solarbio) as the instructions provided by the manufacturer. For hepatocyte Oil red O staining, AML-12 cells were fixed in 4% paraformaldehyde for 30 min at room temperature, then stained with the oil red O staining solution for 15 min and rinsed with PBS.

### Periodic acid-Schiff staining

Mouse liver tissues were fixed in a 10% neutral formalin solution. Following fixation, the tissues were processed for paraffin embedding and sectioned into thin slices using standard histological techniques. The paraffin sections were then subjected to Periodic Acid-Schiff (PAS) staining according to the manufacturer’s protocol provided with the staining kit (#S1281, Solairbio). The positive areas of PAS staining were quantified using Image J software.

### Quantitation of adipocyte size

Tissue sections from epididymal adipose tissue were stained with hematoxylin and eosin (H&E). Image J software was used to measure the adipocyte area, which is represented as the average adipocyte area (in µm^2^). Adipocyte size was measured from five mice per group (> 300 cells/group).

### Immunohistochemistry staining

The mouse liver paraffin sections were stained with anti-ENPP1 antibody (1:1000, #ab314551, Abcam) by immunohistochemistry (IHC) according to the manufacturer’s protocol. The sections were then incubated with Harris hematoxylin for 1 min, rinsed with tap water for 10 min, and subsequently dehydrated and sealed.

### RNA sequencing

Total RNA was extracted, and cDNA libraries were constructed for profiling of gene expression differences. DESeq2 was used to calculate differential gene expression. Genes with a|log2FC|>1 and a P-value < 0.05 were defined as differentially expressed genes (DEGs). Kyoto Encyclopedia of Genes and Genomes (KEGG) pathway annotations for all genes in the reference genome were downloaded from the KEGG database. Pathways with P*-*values < 0.05 were defined as significantly enriched pathways.

### Gene expression omnibus (GEO) database mining

Raw data were deposited in the GEO database (https://www.ncbi.nlm.nih.gov/geo/) under the accession number of GSE126848. Genes showing|log2 fold change| > 0.5 and adjusted P values < 0.05 were considered differentially expressed.

### Cell treatment and transfection

AML-12 cells (normal mouse hepatocytes) were cultured in DMEM/F12 medium (#ZQ606, Shanghai Zhong Qiao Xin Zhou Biotechnology Co.,Ltd.), supplemented with 10% FBS, 100 U/ml penicillin and streptomycin. The cells were plated at a density of 3 × 10^5^ cells on 6-well collagen (Sigma) coated plates and transfected with 100 nM Enpp1-siRNA (F:5’-GUCUCAGUGUCCAAUCAAATT-3’; R:5’-UUUGAU-.

UGGACACUGAGACTT-3’) or 4 µg/µl Enpp1 overexpression plasmid according to the manufacturer’s instructions in normal culture medium for 24 h. Subsequently, the cells were treated with 200 µM palmitic acid (PA) for another 24 h to construct the MAFLD cell model according to previous methods [15].

### Measurement of the AMP-to-ATP ratio in cells

Intracellular AMP and ATP were extracted according to previously reported methods [16]. Then the concentration of AMP and ATP was measured with corresponding assay kits (#V5011, Promega and #S0026, Solarbio) according to the manufacturer’s protocol.

### Statistical analysis

The statistical data were analyzed by using GraphPad Prism 9. Comparisons between two groups were performed using an unpaired two-tailed Student’s t test. A one-way analysis of variance (ANOVA) was used to compare multiple groups,. The data were expressed as the mean ± SEM. *P* < 0.05 was considered statistically significant.

## Results

### Hepatic Enpp1 expression levels are decreased in patients with MAFLD and mouse models

**Fig. 1:**
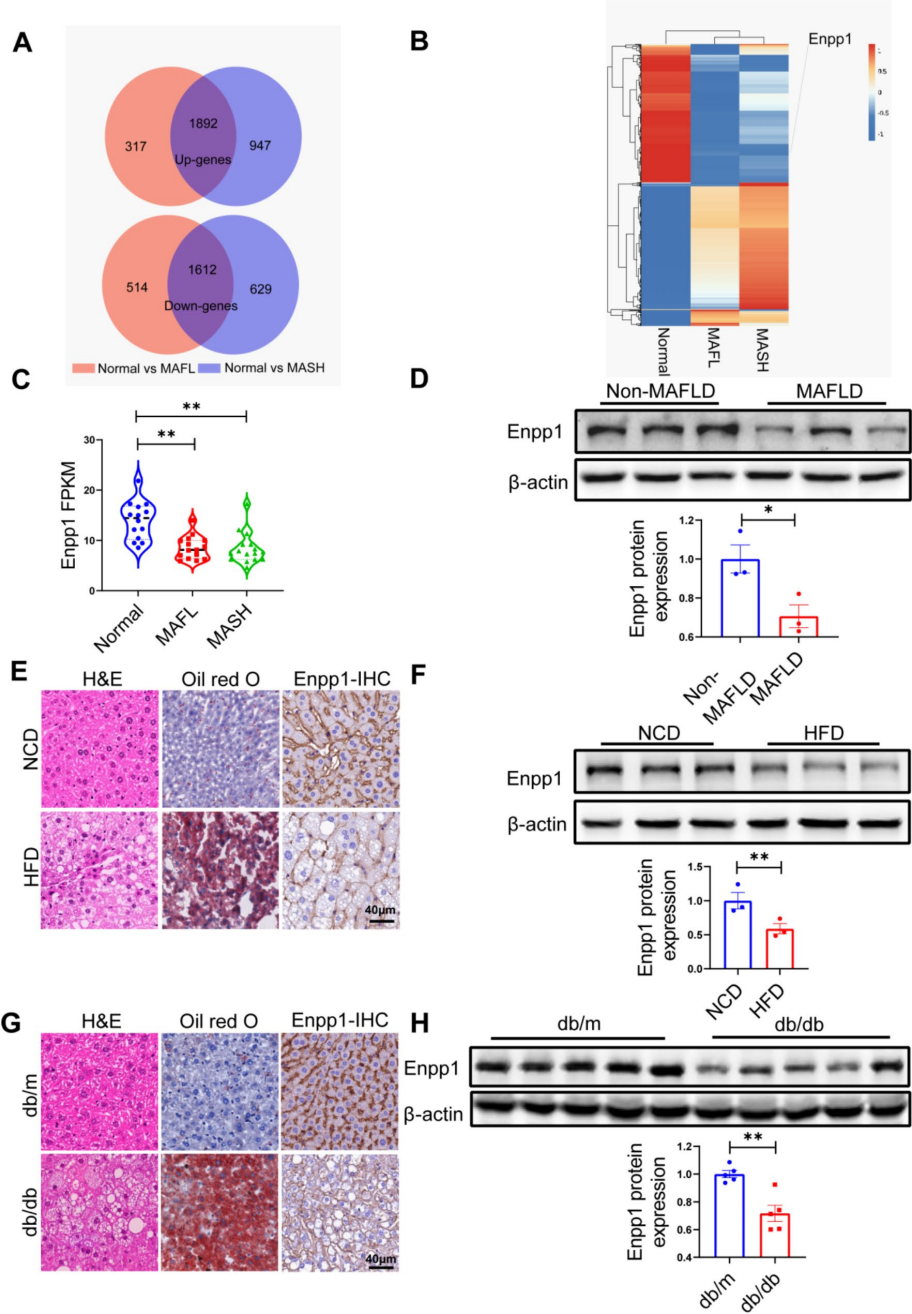
Enpp1 is downregulated in the liver tissue of MAFLD patients and animal models. **(A)** Significantly upregulated (up) and downregulated (bottom) genes in NAFL and NASH patients compared to Normal volunteers. **(B)** Heatmap of co-upregulated and co-downregulated genes in NAFL and NASH. **(C)** Fragments Per Kilobase Million (FPKM) value of ENPP1 expression across volunteer groups. **(D)** Western blot analysis of the expression level of Enpp1 in MAFLD patients, *n* = 3. **(E)** H&E, Oil red O and Enpp1-IHC staining of hepatic speciens from C57BL/6J mice with a normal chow diet (NCD) or high-fat diet (HFD). Scale bar, 40 μm. **(F)** Western blot analysis of Enpp1 in NCD-fed or HFD-fed mice, *n* = 3. **(G)** Representative images of H&E staining, Oil red O staining and Enpp1-IHC staining of liver tissues from db/db or db/m mice. Scale bar, 40 μm. **(H)** Western blot analysis of Enpp1 in db/db and db/m mice, *n* = 5. The datas are expressed as the mean ± SEM, *p < 0.05, **p < 0.01 versus the respective controls

To investigate the genetic basis of the progression of MAFLD, a human RNA sequencing (RNA-seq) dataset for liver samples from diverse conditions including normal, Metabolic dysfunction-associated fatty liver (MAFL), and Metabolic dysfunction-associated steatohepatitis (MASH) were retrieved from the Gene Expression Omnibus (GEO) database. The genes underlying the progression of MAFLD were selected based on the criteria of DEGs (|log2 fold change| > 0.5 and adjusted P value < 0.05). Those that met these thresholds were considered to be significantly different between the MAFL/MASH group and control group. After overlapping, 1892 up-regulated genes and 1612 down-regulated genes were identified in MAFL and MASH respectively (Fig. 1A). Among these DEGs, the Enpp1 mRNA, a novel lipid metabolism biomarker [17], was decreased in liver tissues form MAFL and MASH groups relative to the levels of normal liver tissues (Fig. 1B, C). Western blotting analysis demonstrated lower Enpp1 protein expression levels in MAFLD liver tissues compared with paired normal liver tissues (Fig. 1D). Corresponding experiments were conducted to explore the protein expression of Enpp1 in liver tissues of high-fat diet (HFD) induced MAFLD mouse models and MAFLD gene models (db/db mice). The results demonstrated that the Enpp1 expression was significantly lower in the MAFLD models (Fig. 1E, F) and in db/db obese mice models (Fig. 1G, H). These data demonstrate that Enpp1 plays a crucial role in MAFLD progression.

### Hepatic Enpp1 deficiency aggravates obesity and insulin resistance in mice fed an HFD

**Fig. 2:**
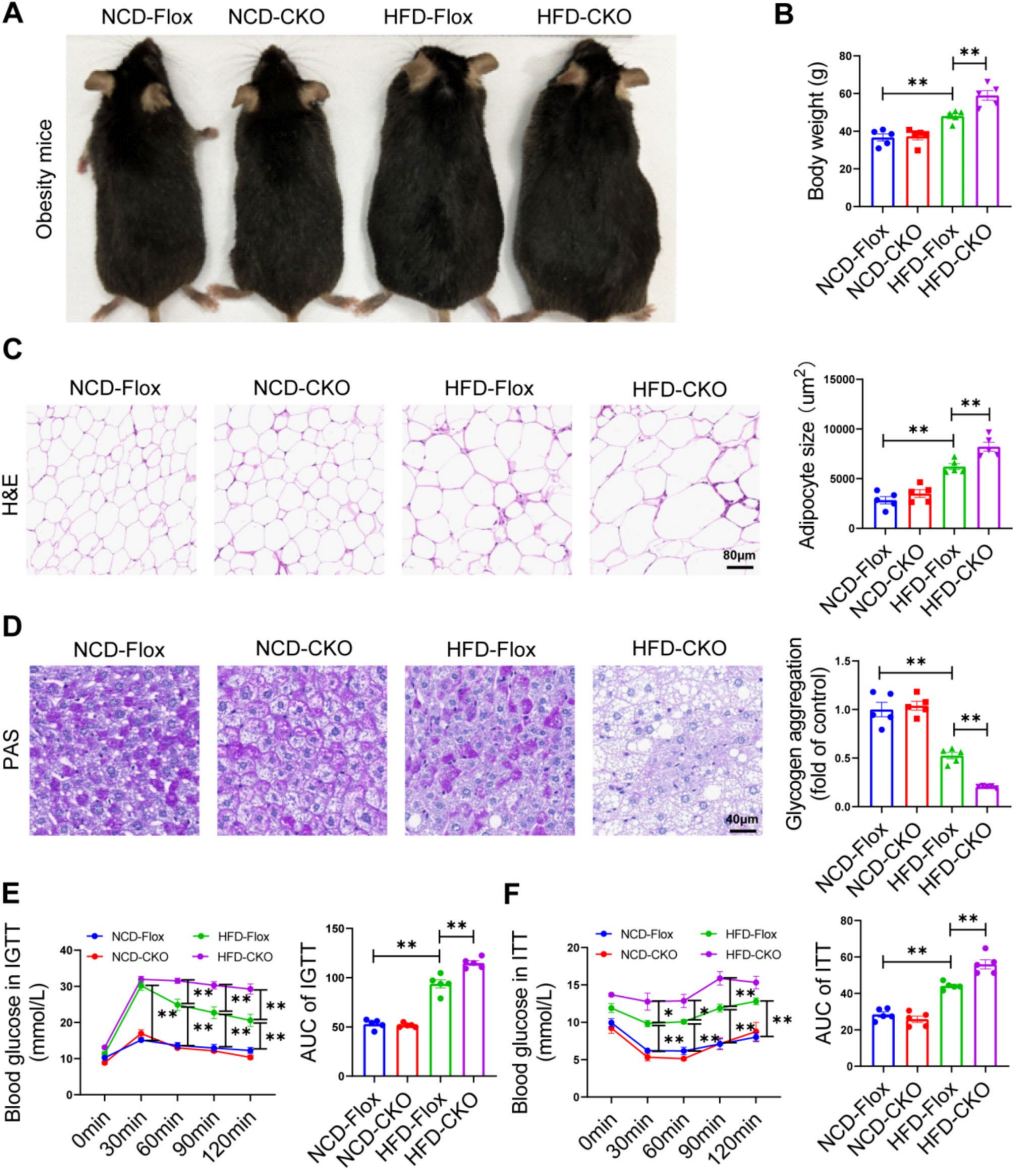
Hepatic Enpp1-knockout exacerbates obesity and insulin resistance induced by HFD in mice. **(A)** Images of HFD-induced obese mice. **(B)** Body weight. **(C)** Representative images of H&E staining of mouse epididymal fat tissue and the average size of adipocytes. Scale bar, 80 μm. **(D)** Representative images of liver Periodic Acid-Schiff (PAS) staining and the percentage of positively stained area. Scale bar, 40 μm. **(E)** Blood glucose levels of mice from the indicated groups subjected to intravenous glucose tolerance test (IGTT) and the corresponding area under curve (AUC) of each group. **(F)** Blood glucose levels of mice in the indicated groups subjected to the insulin tolerance test (ITT) and the corresponding AUC of each group. The datas are expressed as the mean ± SEM, *n* = 5 per group, *p < 0.05, **p < 0.01 versus the respective controls

To further investigate the effects of Enpp1 on MAFLD progression, we established a mouse model with konckout of the Enpp1 gene specifically in the liver. MAFLD model was induced by feeding liver-specific Enpp1 knockout (CKO) mice and the control (Flox) mice with high-fat diet (HFD) or normal chow diet (NCD) for 16 weeks. There was no difference in food intake between CKO mice and Flox mice (Fig. S1). Compared with the HFD-fed Flox mice, the HFD-fed CKO mice developed significant obesity (Fig. 2A), accompanied with increased body weight (Fig. 2B) and larger adipocyte size in the epididymal fat by Hematoxylin-eosin (H&E) staining (Fig. 2C). Periodic acid-Schiff (PAS) staining of mouse livers revealed significant decrease in glycogen content in HFD-fed CKO mice (Fig. 2D), and the expression levels of gluconeogenesis genes increased in the livers of HFD-fed CKO mice (Fig. S2A). Compared with the HFD-fed Flox mice, the HFD-fed CKO mice exhibited significantly higher fasting blood glucose levels (Fig. S2B). Further analysis revealed that the HFD-fed CKO mice showed a greater areas under the curve (AUC) for intravenous glucose tolerance test (IGTT) (Fig. 2E) and insulin tolerance test (ITT) (Fig. 2F). Collectively, our findings suggest that liver-specific Enpp1 knockout in mice fed an HFD exhibited exacerbated obesity and insulin resistance.

### Hepatic Enpp1 deletion exacerbates hepatic steatosis in mice with MAFLD

**Fig. 3:**
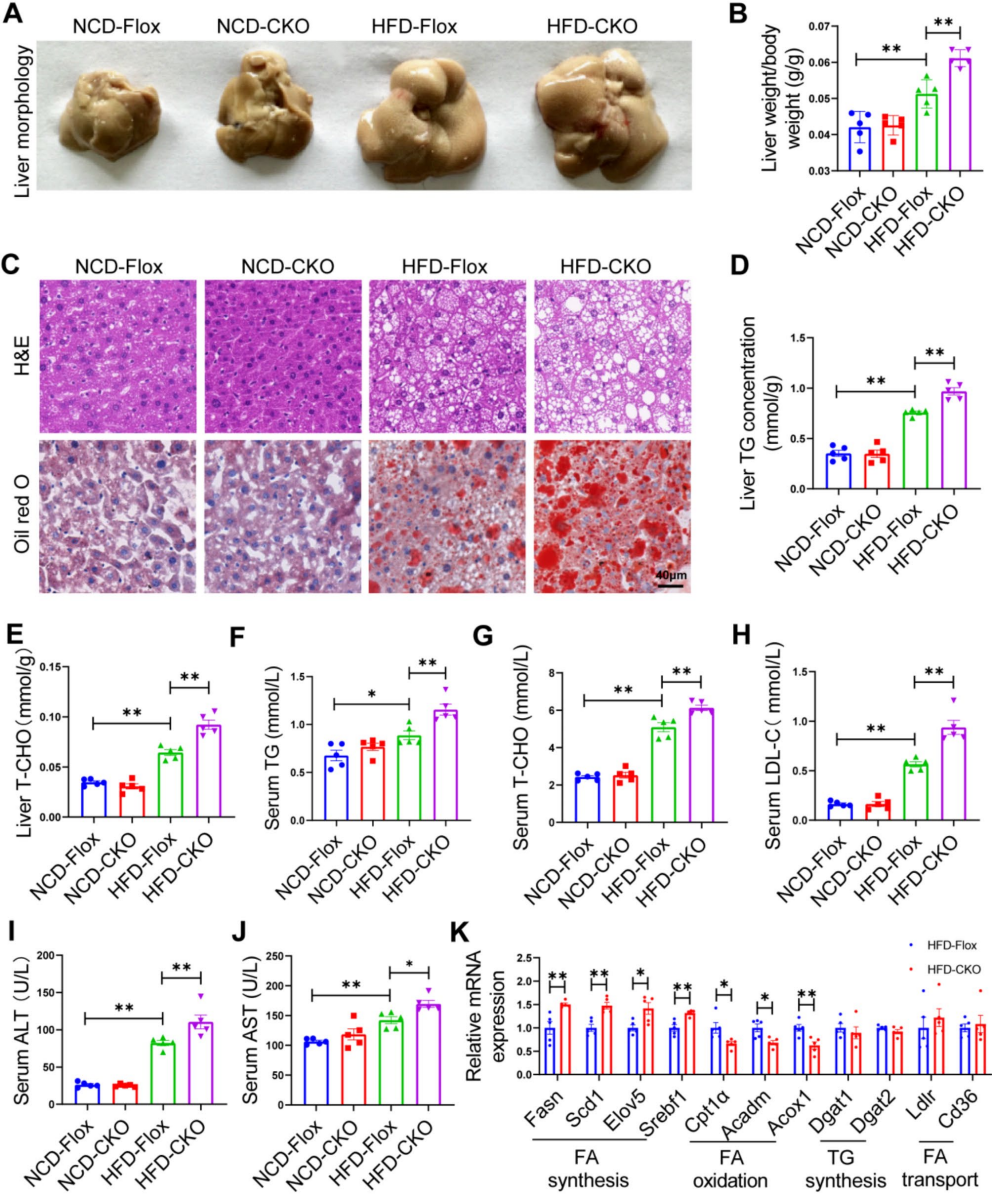
Hepatic Enpp1-knockout mice exhibit increased hepatic steatosis in HFD-induced mouse model of MAFLD. **(A)** Representative liver images of the different groups. **(B)** Liver weight/body weight. **(C)** Representative images of H&E staining (top) and Oil red O staining (below) of liver tissue. Scale bar, 40 μm. **(D)** Liver triglyceride (TG) levels. **(E)** Liver total liver cholesterol (T-CHO) levels. **(F) S**erum TG levels. **(G)** Serum T-CHO levels. **(H)** Serum LDL-C levels. **(I)** Serum ALT levels and **(J)** serum AST levels in control (Flox) and liver-specific Enpp1 knockout (CKO) mice after 16 weeks with NCD or HFD diet. **(K)** qPCR analysis of the mRNA levels of lipid metabolism-related genes in each group. The datas are expressed as the mean ± SEM, *n* = 5 per group, *p < 0.05, **p < 0.01 versus the respective controls

Furthermore, we established liver-conditional knockout of Enpp1 (CKO) mice to elucidate the effect of Enpp1 on hepatic steatosis. CKO mice fed with an HFD for 16 weeks, exhibited increased liver weight/body weight and liver steatosis but not those fed with an NCD (Fig. 3A-C). Moreover, HFD increased the concentration of liver triglycerides (TG) and liver total cholesterol (T-CHO) relative to the controls (Fig. 3D, E). Additionally, serum TG, T-CHO and low-density lipoprotein cholesterol (LDL-C) levels were increased in HFD-fed CKO group than in the HFD-fed Flox group, suggesting the CKO mice had enhanced HFD-induced hepatocellular lipid accumulation (Fig. 3F-H). Morever, serum alanine transaminase (ALT) and aspartate aminotransferase (AST) levels were significantly elevated in the HFD-fed CKO group, indicating that the HFD-induced hepatocyte damage was aggravated in the CKO mice (Fig. 3I, J). To elucidate the effect of Enpp1 on lipid metabolism, detected the mRNA levels of hepatic regulators of fatty acid synthesis (Fasn, Scd1, Elov5 and Srebf1), fatty acid oxidation (Cpt1α, Acadm and Acox1), TG synthesis (Dgat1 and Dgat2) and fatty acid transportation (Ldlr and Cd36). It was observed that, under HFD conditions, the expression of the genes involved in fatty acid synthesis was up-regulated, while that of genes involved in fatty acid oxidation were decreased in the livers of CKO mice (Fig. 3K). Altogether, the present findings suggest that hepatic Enpp1 deficiency exacerbated hepatic steatosis progression.

### Enpp1 overexpression alleviates hepatic steatosis and metabolic deterioration

**Fig. 4:**
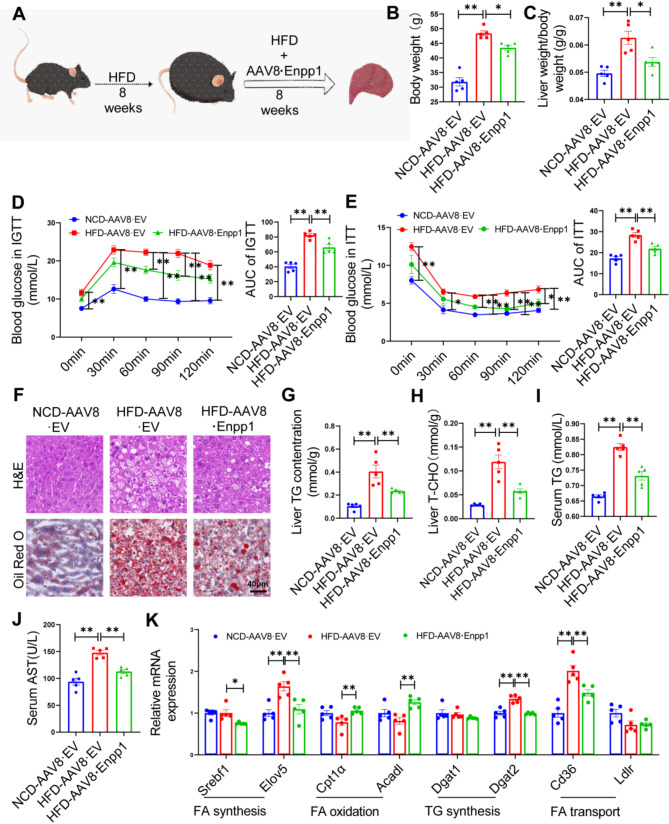
Enpp1 treatment protects against insulin resistance and hepatic steatosis in HFD-fed mice. **(A)** Schematic of the experimental procedure to test the role of Enpp1 in HFD-induced MAFLD. **(B)** Body weight and **(C)** liver weight/body weight at 16 th week on diet. **(D)** Blood glucose levels of mice in the indicated groups subjected to IGTT (14 th week on treatment), and area under the curve of IGTT performed on mice treated with or without AAV8·Enpp1. **(E)** Blood glucose levels of mice in the indicated groups subjected to ITT (15 th week on treatment) and area under the curve of ITT on mice treated with or without AAV8·Enpp1. **(F)** Representative histology of H&E staining (up) and Oil red O staining (below) on liver sections. Scale bar, 40 μm. **(G-J)** Endpoint (16 th week on a diet) measurements of liver TG **(G)**, liver T-CHO **(H)**, serum TG **(I)** and AST levels **(J)**. **(K)** Genes related to lipid metabolism expression normalized to β-actin. The datas are expressed as the mean ± SEM, *n* = 5 per group, *p < 0.05, **p < 0.01 compared to respective controls

Based on the above results, we further analyzed the impact of hepatic Enpp1 overexpression on hepatic steatosis. Briefly, wild-type C57BL/6J mice (8 weeks of age) were fed with either an NCD or an HFD for 8 weeks. Subsequently, the HFD-fed mice were administered with empty vector adenovirus (AAV8·EV, 2 × 10^12^ genome copies per mouse) or the Enpp1 liver-specific overexpression adenovirus (AAV8·Enpp1, 2 × 10^12^ genome copies per mouse) via the tail vein on the 8th week followed by further feeding for 8 weeks (Fig. 4A). The results indicated that HFD-fed mice overexpressing Enpp1 underwent a reduction in body weight and liver weight/body weight compared with vehicle-treated HFD-fed mice (Fig. 4B, C). Moreover, Enpp1 overexpression improve glucose tolerance (Fig. 4D) and insulin resistance (Fig. 4E) in mice. Histochemical analysis based on H&E and Oil red O staining revealed that Enpp1 overexpression reduced hepatic steatosis (Fig. 4F), which was consistent with the reduction in liver TG levels, liver T-CHO levels and serum TG levels in Enpp1-overexpressed HFD-fed mice (Fig. 4G-I). Importantly, Enpp1 overexpression resulted in a significant decrease in serum AST levels (Fig. 4J), indicating a reduction in liver damage. In addition, Enpp1 overexpression under HFD conditions decreased the levels of key hepatic regulators of fatty acid synthesis (Srebf1 and Elov5), TG synthesis (Dgat2) and fatty acid transportation (Cd36), but increased the expression of Cpt1α and Acadl, which are rate-limiting enzymes during fatty acid oxidation (Fig. 4K). Collectively, these findings indicated that Enpp1 exerted its protective effects in vivo by down-regulating lipid synthesis and enhancing fatty acid oxidation, to mitigate MAFLD progression.

### Enpp1 regulates hepatic lipid metabolism through activating the AMPK/PPARα signaling pathway

**Fig. 5:**
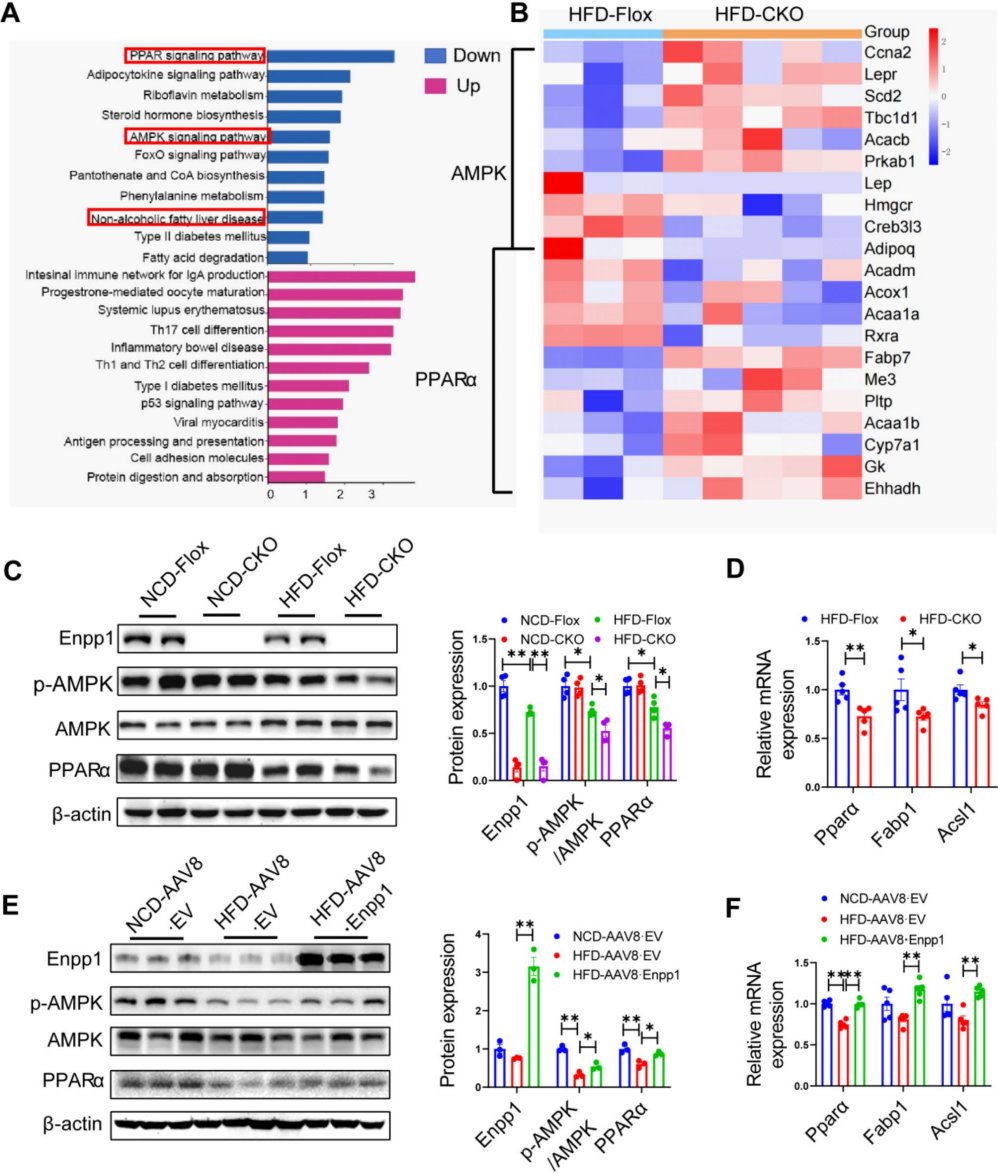
The AMPK and PPARα signaling pathways are potential signaling pathways regulated by Enpp1. **(A)** KEGG pathway enrichment analysis of the HFD-fed CKO group and control group. **(B)** Differential gene enrichment thermogram of the AMPK and PPARα pathways. **(C)** Representative western blots showing the AMPK and PPARα signaling pathways were inhibited after the deletion of Enpp1 in HFD fed mice. **(D)** Downstream gene expression of PPARα determined via qPCR. **(E)** Representative western blots of AMPK and PPARα signaling pathways were activated after the overexpression of Enpp1 in HFD fed mice. **(F)** Downstream gene expression of PPARα determined by qPCR after Enpp1overexpression treatment. The datas are expressed as the mean ± SEM, *n* = 3–5 per group. *p < 0.05, **p < 0.01 versus the respective controls

To explore the molecular mechanisms mediating the effects of Enpp1 on lipid metabolism, RNAseq data from liver samples of HFD-fed CKO mice and HFD-fed Flox mice were analyzed via Kyoto Encyclopedia of Genes and Genomes (KEGG) pathway enrichment. The analyses showed that differentially expressed genes were strongly associated with the lipid metabolism pathway, including peroxisome the proliferator-activated receptor α (PPARα) and AMP-activated protein kinase (AMPK) (Fig. 5A, B). Further, we investigated that correlation between Enpp1 and the AMPK/PPARα pathway, and uncovered that the phosphorylation of AMPK and PPARα expression were significantly decreased in the liver of HFD-fed CKO mice (Fig. 5C). And, the expression levels of downstream target genes of PPARα (Fabl1 and Acsl1) in the liver of HFD-fed CKO mice was significantly lower compared with levels in the HFD-fed Flox mice, which indicated that fatty acid oxidation in the liver was inhibited (Fig. 5D). In contrast, Enpp1 overexpression induced AMPK phosphorylation and PPARα expression (Fig. 5E), as well as the downstream target genes of PPARα (Fig. 5F). These findings suggest that Enpp1 inhibits lipid synthesis by enhancing the activation of AMPK/PPARα, thereby exerting a protective effect *in vivo.*

### Enpp1 activates the AMPK/PPARα axis by increasing the AMP-to-ATP ratio to inhibits TG accumulation in hepatocytes

**Fig. 6:**
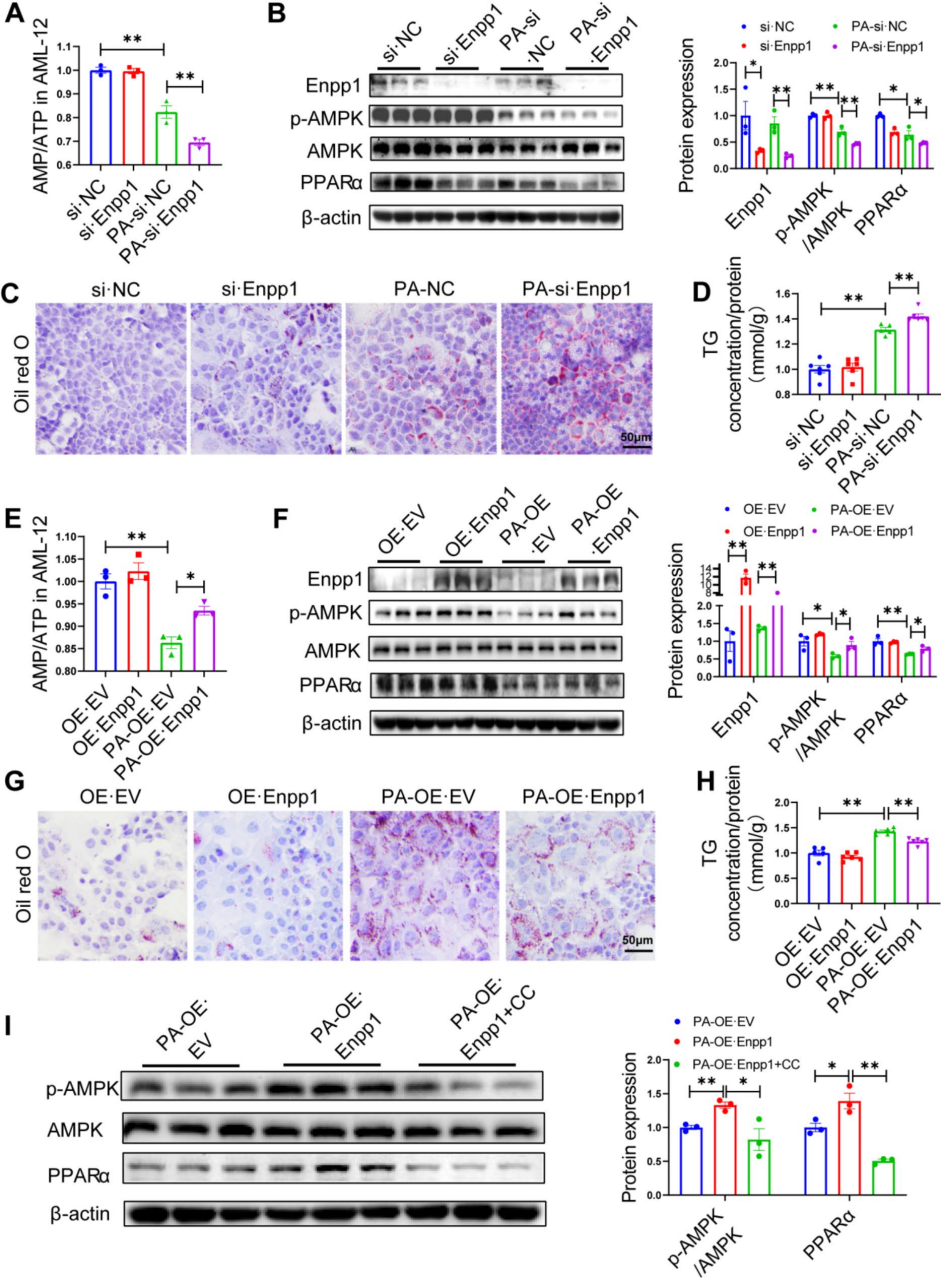
Enpp1 inhibits lipid deposition by activating the AMPK/PPARα axis through modulating the AMP-to-ATP ratio. AML-12 cells were transfected with Enpp1-siRNA (si·Enpp1), Control-siRNA (si·NC), the Enpp1 overexpression plasmid (OE·Enpp1) or the empty vector (OE·EV) and then cotreated with or without PA (200 µM). **(A)** AMP-to-ATP ratio in AML-12 cells after Enpp1 is downregulated. **(B)** Western blotting was used to detect the Enpp1 protein level and AMPK/PPARα signaling pathway with Enpp1 was slienced. **(C)** Intracellular lipid content, Scale bar, 50 μm. **(D)** Intracellular triglyceride content. **(E)** AMP-to-ATP ratio in AML-12 cells after Enpp1 is up-regulated. **(F)** Western blotting was used to detect the Enpp1 protein level and AMPK/PPARα signaling pathway with Enpp1 was overexpressed. **(G)** Intracellular lipid level, Scale bar, 50 μm. **(H)** Intracellular triglyceride content. **(I)** Representative western blots of the indicated proteins in the presence or absence of compound C (CC, 20 µM) treatment. The datas are expressed as the mean ± SEM, *n* = 3–6 per group, *p < 0.05, **p < 0.01 versus the respective controls

To further verify the regulatory of Enpp1 on the expression of AMPK/PPARα, we conducted in vitro experiments using siRNA and plasmid transfection to knockdown or overexpress Enpp1 in AML-12 cells (normal mouse hepatocytes) treated with PA. Considering the role of Enpp1 in hydrolysing ATP into AMP and PPi, and that the AMP-to-ATP ratio is the core factor modulating AMPK phosphorylation [18, 19], we speculated that Enpp1 may activate the AMPK/PPARα signaling pathway by regulating the AMP-to-ATP ratio. Notably, Enpp1 knock down resulted in significant reduction in AMP-to-ATP ratio (Fig. 6A), expression of phosphorylated AMPK and PPARα (Fig. 6B), and expression of the PPARα downstream genes in AML-12 cells treated with PA (Fig. S3A). It was also observed that Enpp1 knock down significantly increased the lipid droplets and TG content in AML-12 cells (Fig. 6C, D). This was opposite the effects of Enpp1 overexpression, in which the increase in Enpp1 expression increased the AMP-to-ATP ratio (Fig. 6E) and enhanced the AMPK phosphorylation and PPARα protein expression as well as the downstream genes of PPARα in AML-12 cells treated with PA (Fig. 6F and Fig. S3B). In additon, Enpp1 overexpression suppressed lipid accumulation and TG levels in AML-12 cells treated with PA (Fig. 6G, H). To confirm the effect of AMPK modification on the crosstalk between Enpp1 and PPARα, we introduced a small-molecule inhibitor of AMPK, namely, compound C (CC). Specifically, the AML-12 cells transfected with Enpp1 overexpression plasmid were divided into the control group (DMSO for 24 h) and the CC group (20 µM for 24 h). Subsequently, the protein expression of AMPK phosphorylated and PPARα was detected by western blot analysis. The results showed that CC intervention decreased the expression levels of these proteins compared with the control group, which suggested that CC blocked the protein synthesis of PPARα by inhibiting Enpp1-mediated AMPK (Fig. 6I). These findings demonstrated that Enpp1 activated the AMPK/PPARα signaling pathway by increasing the AMP-to-ATP ratio, thus alleviating lipid accumulation in hepatocytes.

## Disscussion

The MAFLD disorder, previously known as nonalcoholic fatty liver disease, is a disease characterized by fat accumulation in the liver accompanied with metabolic dysfunction including obesity and insulin resistance [20, 21]. Currently, the role of Enpp1, a key protein closely associated with MAFLD progression, in the hepatic steatosis and metabolic dysregulation of MAFLD is not well understood. A previous study demonstrated that Enpp1 gene polymorphisms increase the risk of maternal and neonatal obesity [17]. Findings from another investigation showed that overexpression of Enpp1 in subcutaneous adipose tissue contributed to the development of obesity [22]. A recent study confirmed that Enpp1 was either overexpressed or overactive in muscles, adipose tissues, fibroblasts and other tissues of non-diabetic and diabetic insulin-resistant individuals [20]. Elsewhere, it was uncovered that mice with impaired glucose tolerance showed decreased liver Enpp1 protein expression in the context of high circulating insulin level [22]. In this study, we analyzed the expression of Enpp1 in the public databases and validated it through western blotting analysis of clinical specimens from MAFLD patients. We found that Enpp1 was downregulated in MAFLD.

To determine the effect of Enpp1 on hepatic steatosis and metabolic dysregulation in MAFLD, we developed liver-specific knockout of Enpp1 (CKO) mice Enpp1 and liver-specific overexpression of Enpp1 mice. In addition, i*n vivo* experiments were performed in which Enpp1 was knocked down. It was observed that Enpp1 knockout decreased decreased insulin sensitivity under the high-fat diet (HFD) condition, but it slightly improved insulin sensitivity under normal chow diet (NCD) condition. Previous studies demonstrated that impaired insulin sensitivity induced abnormal increase in hepatic gluconeogenesis [23], whereas Enpp1 deletion upregulated the levels of gluconeogenic genes and decreased liver glycogen levels. These results indicate that Enpp1 can alleviate the MAFLD induced by obesity and insulin resistance, however the causal relationship among obesity, insulin resistance and MAFLD regulated by Enpp1 remains to be further investigated. Additionally, we observed that HFD fed liver-specific Enpp1 knockout (CKO) mice had significantly worse hepatic steatosis, wheres specific those with liver-specific overexpression of Enpp1 had reduced liver lipid accumulation. Notably, there were no phenotypic differences in mice with NCD-fed CKO compared with the normal mice, which lead us to speculated that the lack of high fat and other inducing factors during normal diet feeding, so it has no obvious metabolic impact on the body. Although we also observed that the triglyceride content in the liver and serum of NCD-fed CKO mice has an upward trend. This finding suggest that Enpp1 knockout can cause high-risk state of MAFLD. In addition, we found that HFD-fed CKO mice group had more severe MAFLD than the control group, which further suggest the important role of Enpp1 in the steady-state regulation of lipid metabolism.

Through deep mining of RNAseq data from liver samples of HFD-fed CKO mice and HFD-fed Flox mice, we identified the AMP-activated protein kinase (AMPK) and proliferator-activated receptor α (PPARα ) as the targets genes of Enpp1 associated with the lipid metabolism pathway. Further experiments showed that HFD-fed CKO mice group had significantly lower levels of phosphorylated of AMPK and PPARα in the liver. AMPK, as an important kinase that regulates energy homeostasis, is one of the central regulators of metabolism in eukaryotic cells and organisms, with previous studies demonstrating that it modulates the expression of PPARα [24]. The latest research has demonstrated that Enpp1 deficiency may influence the development of knee OA by inhibiting the AMPK signaling pathway, but the mechanisms involed were not clarified [25]. In this study, we attempted to explore the specific mechanism. Intracellular AMP is the main activator of AMPK [26, 27], which is produced during the hydrolysis of intracellular ATP and other purine nucleotides in various physiological processes [28]. Given that Enpp1 has been shown to regulate hydrolysis of different purine nucleotides in diverse physiological processes, including the hydrolysis of ATP to AMP [28, 29], we used AML-12 cells to estimate AMP-to-ATP ratio. Knock down of Enpp1 expression in AML-12 cells treated with PA resulted in a significant decrease in the AMP-to-ATP ratio, a significant reduction in AMPK phosphorylation and PPARα protein levels, and an increase in intracellular lipid accumulation and triglyceride content. Conversely, Enpp1 overexpression increased the AMP-to-ATP ratio, AMPK phosphorylation and PPARα protein levels, but decreased the intracellular triglyceride content. This was similar to the results obtained in plasma AMP-to-ATP ratio in patients with Enpp1 deficiency [30, 31]. These experiments reveal that Enpp1 activates AMPK/PPARα pathway by increasing AMP-to-ATP ratio, to modulate lipid metabolism.

This study has some limitations that should be acknowledged. Firstly, we did not deeply investigate the specific effect of Enpp1 on lipid metabolism via the AMPK/PPARα pathway. However, previous studies have reported that activation of parenchymal cell PPARα improves hepatic lipid metabolism by increasing ‌ω-oxidation as well as peroxisomal and mitochondrial β-oxidation [32]. Although the effects of Enpp1 on lipid metabolism of MAFLD were demonstrated, further investigation are needed to determine its effects on immune phenotypes. MAFLD is often accompanied by an increase of inflammatory mediators (including cytokines and chemokines), which increases intrahepatic infiltration of various types of immune cells [31, 33]. Enpp1, as an extracellular enzyme, can hydrolyze extracellular cGAMP, thereby inhibit the STING pathway and inhibit the production of Interferon type I (IFN-1) and other pro-inflammatory cytokines [34]. Enpp1 can also contribute to the occurrence and development of hepatocellular carcinoma by regulating the infiltration of NK cells, dendritic cells and Th17 immune cells [17]. Other studies have shown that Enpp1 may exert inflammatory effects during the progression of MAFLD/MASH disease, which requires further confirmation.

## Conclusions

In summary, we provides both clinical and experimental evidence that Enpp1 participates in the progression of MAFLD. Enpp1 deficiency in liver results in abnormal lipid accumulation, which accelerates the development of MAFLD. Overexpression of Enpp1 inhibits lipid accumulation in hepatocytes to ameliorate MAFLD. Mechanistically, Enpp1 activates the AMPK/PPARα signaling by increasing AMP-to-ATP ratio to reduce lipid content in hepatocytes. Therefore, this study not only uncovered a novel molecular mechanism of MAFLD progression but also identified the Enpp1 protein as a potential new molecular target for the clinical management of MAFLD.

## Electronic supplementary material

Below is the link to the electronic supplementary material.

Supplementary Material 1

Supplementary Material 2

## Data Availability

The data that support the findings in this study are available in the manuscript and Supplementary Materials of this article. The RNA-Seq datasets generated during the current study were submitted to Genome Sequence Archive (GSA). All other relevant data are available from the corresponding author on request.

## Abbreviations

MAFLD: Metabolic dysfunction associated fatty liver disease
HFD: High fat diet
NCD: Normal Chow Diet
TG: Triglyceride
T-CHO: Total Cholesterol
AMPK: AMP-activated protein kinase
p-AMPK: Phospho-AMP-activated protein kinase
PPAR: Peroxisome Proliferator-Activated Receptor
AMP: Adenosine Monophosphate
ATP: Adenosine Triphosphate
H&E: Hematoxylin and Eosin
PAS: Periodic Acid-Schiff
CKO: Conditional Knockout
OE: Over Expression
EV: Empty Vector
KEGG: Kyoto Encyclopedia of Genes and Genomes
PPi: Pyrophosphate
DEGs: Different Expressed Genes
