# Supplementary material for: Enpp1 ameliorates MAFLD by regulating hepatocyte lipid metabolism through the AMPK/PPARα signaling pathway
Source: Cell Biosci. 2025 Feb 19;15:22. doi: 10.1186/s13578-025-01364-3 (PMC11841222; doi:10.1186/s13578-025-01364-3)
Supplement: Supplementary file 1 — Supplementary Material 1 [file 13578_2025_1364_MOESM1_ESM.docx]

**Supplementary Information**

**Enpp1 ameliorates MAFLD by regulating hepatocyte lipid metabolism through the AMPK/PPARα signaling pathway**

**Xiaohui Liu^1^, Shuai Chen^2^, Xing Liu^1^, Xianxian Wu^1^, Xiaoliang Jiang^1^, Yuhan Li^3*^; Zhiwei Yang^1*^**

1. Institute of Laboratory Animal Science, Chinese Academy of Medical Sciences (CAMS) & Comparative Medicine Centre, Peking Union Medical College (PUMC), Beijing, China

2. Fuyang People's Hospital affiliated to Anhui Medical University，Fuyang, China

3. Department of Clinical Laboratory, Beijing Friendship Hospital, Capital Medical University, Beijing, China

****Correspondence authors:****

Yuhan Li, Department of Clinical Laboratory, Beijing Friendship Hospital, Capital Medical University, Beijing 100050, China. Email: liyuhan2455@163.com

Zhiwei Yang, Institute of Laboratory Animal Science, Chinese Academy of Medical Sciences (CAMS) & Comparative Medicine Centre,Peking Union Medical College, Beijing, 100021, China. Email:yangzhiwei@cnilas.pumc.edu.cn

**Supplementary Figures**


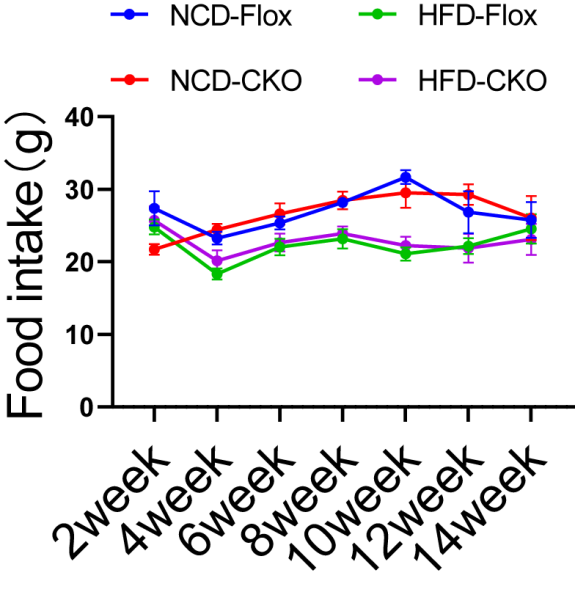


**Fig. S1** Dietary intake among corresponding groups of mice. The average feed consumption weight of each cage of mice is counted every two weeks, and the count continues until the 14 th week, with the number of mice in each cage was 5.


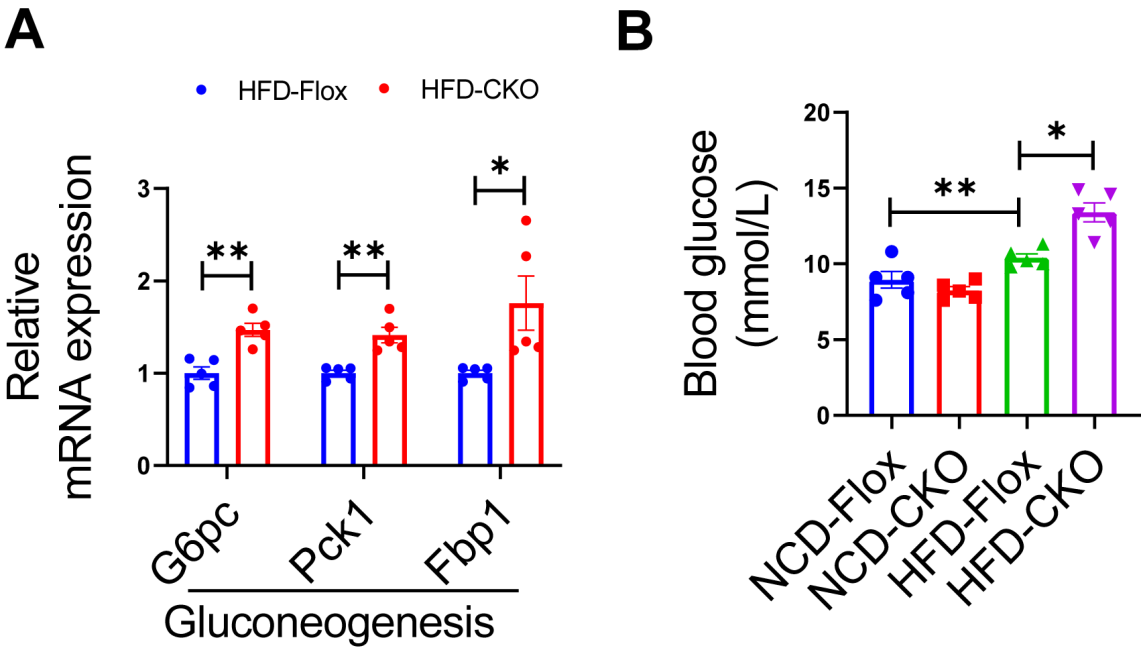


**Fig. S2** Detection of Hepatic Gluconeogenesis in Mice. A) qPCR analysis of the expression levels of gluconeogenesis-related genes. B) Fasting blood glucose after 6 h of fasting. n = 5*,* the datas are expressed as the mean ± SEM, *p < 0.05, **p < 0.01 versus the respective controls.


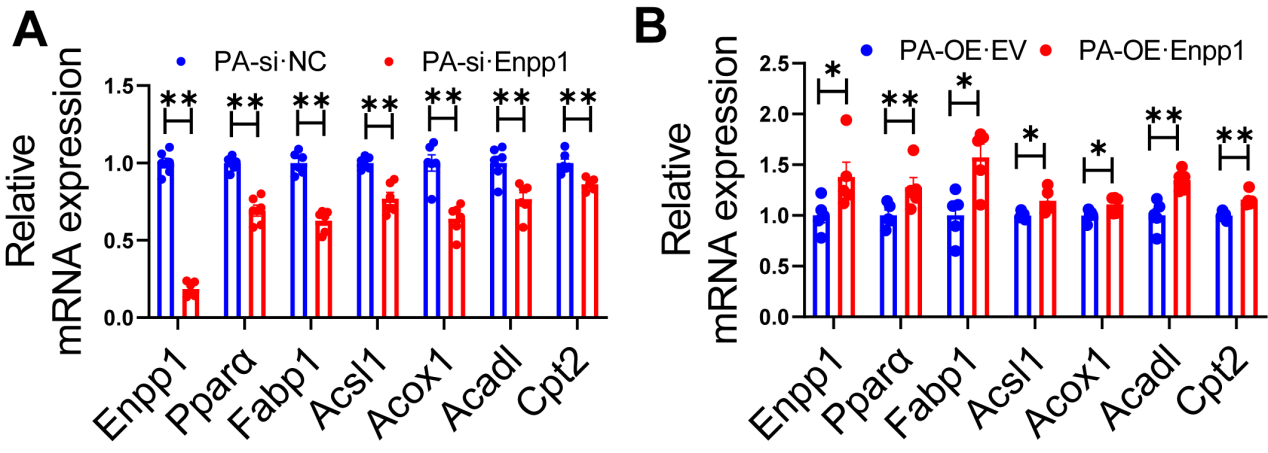


**Fig. S3** The expression of PPARα downstream genes following Enpp1 was modulated in AML12 cells. A) Downstream gene expression of PPARα after Enpp1 knockdown determined by qPCR. B) Downstream genes expression of PPARα after Enpp1 over-expressed determined via qPCR. The data are expressed as the mean ± SEM (n = 3-6 per group). *p <0.05, **p <0.01 versus the respective controls.

**Supplementary Table**

TABLE S1 Primer sequences

| Gene | Species | Sequence | |
| --- | --- | --- | --- |
| G6pc | Mouse | Forward | TGAGACCGGACCAGGAAGTC |
|  |  | Reverse | GCAAGGTAGATCCGGGACAG |
| Pck1 | Mouse | Forward | ATCCCCAAAACTGGCCTCAG |
|  |  | Reverse | TACATGGTGCGGCCTTTCAT |
| Fbp1 | Mouse | Forward | GCATCGCACAGCTCTATGGT |
|  |  | Reverse | ACAGGTAGCGTAGGACGACT |
| Fasn | Mouse | Forward | GGAGGTGGTGATAGCCGGTAT |
|  |  | Reverse | TGGGTAATCCATAGAGCCCAG |
| Scd1 | Mouse | Forward | GCTGAACACCCATCCCGAG |
|  |  | Reverse | TGTAAGAACTGGAGATCTCTTGGA |
| Elovl5 | Mouse | Forward | TGCAGCTTGCTTCTGTTCCC |
|  |  | Reverse | TTTGACTCTTGTATCTCGGGGGG |
| Srebf1 | Mouse | Forward | ACTTTTCCTTAACGTGGGCCT |
|  |  | Reverse | TGAGCTGGAGCATGTCTTCG |
| Cpt1α | Mouse | Forward | GACTCCGCTCGCTCATTCC |
|  |  | Reverse | GAGATCGATGCCATCAGGGG |
| \| Acadm \| \| --- \| | Mouse | Forward | TCAAGATCGCAATGGGTGCT |
|  |  | Reverse | GCTCCACTAGCAGCTTTCCA |
| Pparα | Mouse | Forward | TGTGAACTGACGTTTGTGGC |
|  |  | Reverse | CCACAGAGCGCTAAGCTGT |
| Dgat1 | Mouse | Forward | TGGTAGTGGGCCCAAGGTAG |
|  |  | Reverse | TGCAGACGATGGCACCTCAG |
| Dgat2 | Mouse | Forward | CTGTCACCTGGCTCAACAGAT |
|  |  | Reverse | CACTGCAGGCCACTCCTAGC |
| Ldlr | Mouse | Forward | CCAATCGACTCACGGGTTCA |
|  |  | Reverse | ACAGTGTCGACTTCTCTAGGC |
| Cd36 | Mouse | Forward | TGTGGAGCAACTGGTGGATG |
|  |  | Reverse | CGTGGCCCGGTTCTAATTCA |
| \| Acadl \| \| --- \| | Mouse | Forward | GTCCGATTGCCAGCTAATGC |
|  |  | Reverse | CACAGGCAGAAATCGCCAAC |
| Acox1 | Mouse | Forward | TGAACAAGACAGAGGTCCACG |
|  |  | Reverse | GATTCGGCCTCTCTGTGGAG |
| Acaca | Mouse | Forward | CCGCCAGCCTGAGTTCTTT |
|  |  | Reverse | TTTGGCCAACGGAGATGGTT |
| Fabp1 | Mouse | Forward | TCCGCAATGAGTTCACCCTG |
|  |  | Reverse | GCTTGACGACTGCCTTGACT |
| Acsl1 | Mouse | Forward | TGCTGTGCACCCGGAATTAT |
|  |  | Reverse | TAGTTCCGTAGCTCTGGCCT |
| Cpt2 | Mouse | Forward | TATCTGCAGCACAGCATCGT |
|  |  | Reverse | GTTTAGGGATAGGCAGCCTGG |
| β-actin | Mouse | Forward | GATTACTGCTCTGGCTCCTAGC |
|  |  | Reverse | GACTCATCGTACTCCTGCTTGC |
